# Estimating Rooting Depth From Herbarium Specimens Might Be More Accurate Than Using Large Trait Databases

**DOI:** 10.1002/ece3.71529

**Published:** 2025-06-17

**Authors:** Attila Takács, Attila Molnár V., Anna E‐Vojtkó, Jenő Nagy

**Affiliations:** ^1^ Department of Botany University of Debrecen Debrecen Hungary; ^2^ HUN‐REN‐UD Conservation Biology Research Group Debrecen Hungary; ^3^ Institute of Botany, Czech Academy of Sciences Třeboň Czech Republic

**Keywords:** bias, functional trait, maximum rooting depth, natural history collection, prediction

## Abstract

Global databases of plant functional traits are facing issues in data heterogeneity and taxonomical or geographical representativeness. To fill data gaps, natural history collections, such as herbaria, have become widely accepted as a potential source of data on functional traits. Surprisingly, root characteristics of plants still have not been studied on herbarium materials. We investigated whether rooting depth data from herbarium samples are realistic enough to be used in ecological studies. We measured original maximum rooting depth records on herbarium specimens and individuals from the field. Global data from the TRY database were also obtained. We tested the pairwise correlations between data from the three datasets. The effect of life form, taxonomic position, and average species height on rooting depth was also evaluated. Herbarium data showed strong correlation to field records, while records from the TRY database showed a weaker correlation with data measured on herbarium materials. Life form, taxonomic position, and height proved to be good predictors of rooting depth collected from the field or the herbarium; however, the model including data obtained from the TRY as the response variable performed weaker. We constructed an equation for predicting realistic average maximum rooting depth values of a given species based on herbarium data. Strong correlation among the field and herbarium datasets suggests that museal collections can be considered as resources of root trait data. Although herbarium‐based rooting depth measurements usually represent lower values than field records, the correction of the herbarium‐derived dataset is solvable. These corrected data might be more accurate than using large, global trait databases. Herbarium work might be a more sustainable, time‐ and cost‐effective practice than field sampling. The inclusion of herbarium‐derived information in trait‐based studies, as well as in global databases, can improve these sources spatially, temporally, and taxonomically.

## Graphical abstract

Herbaria have become widely accepted as a potential source of data on functional traits. We investigated whether rooting depth data from herbarium samples are realistic enough to be used in ecological studies. Although herbarium‐based rooting depth measurements usually represent lower values than field records, after correction, these might be more accurate than the content of global trait databases.

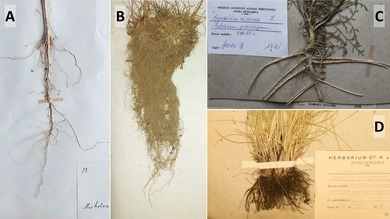

## 1 Introduction

Herbaria traditionally provide materials for taxonomical and biogeographical research. In recent decades, the range of potential usage of these collections has become wider (Funk 2003; Pyke and Ehrlich 2010; James et al. 2018). Closer cooperation between botanists (e.g., herbarium collectors) and ecologists has already been called for by Stern and Eriksson (1996). Since then, many studies have been published, in which information of ecological relevance has been extracted (at least in part) from natural history collections. The scope of these studies is very diverse, including, e.g., timing of life‐history events (e.g., Molnár et al. 2012; Ouédraogo et al. 2020), seed viability (e.g., Godefroid et al. 2011; Molnár et al. 2015), plant–pollinator (e.g., Byers 2017), plant–herbivore (e.g., Meineke and Davies 2019), and plant–fungi interactions (e.g., Antonovics et al. 2003; Nagy et al. 2019). Collection‐based research is greatly encouraged by the increasing digitization of collections (Drew et al. 2017; Soltis 2017; Davis 2023).

As the field of functional ecology has evolved, and the role of a trait‐based approach is growing (McGill et al. 2006; Gallagher et al. 2020), many studies have already considered natural history collections as a potential data source of functional traits. Although large, global trait databases (e.g., TRY: Kattge et al. 2020) are still serving as the primary source of available plant trait data, they are still facing multiple challenges concerning data heterogeneity, completeness (Cornwell et al. 2019) and geographical representativeness (König et al. 2019). Naturally, there are some concerns regarding biases and limitations in the use of herbarium data as well (Kozlov et al. 2020; Perez et al. 2020), however, other authors have argued that this should not exclude the use of these collections as trait data sources (Meineke and Daru 2021; Davis 2023). Heberling (2022) directly states that “herbaria should be embraced as centres for functional trait research”! Heberling (l.c.) illustratively reviewed the usage of herbaria as sources of plant traits and stressed the untapped potential of collections in many respects. Even though some plant functional traits are routinely measured from herbaria, surprisingly, characteristics of underground plant organs have been studied very rarely on herbarium materials. Only a few studies have examined the morphology and anatomy (Price 1911; Schneider 2000) or size (McGraw 2001) of roots in herbarium specimens.

Although knowledge on the function of below‐ground organs has also been developing in recent years, this segment of plant ecology faces several methodological difficulties and conceptual questions (McCormack et al. 2015; Klimešová et al. 2018). Root ecology lags behind, e.g., leaf trait ecology, both conceptually and methodologically (Heberling 2022). The available data pool for root traits—even compared to above‐ground characters—is highly incomplete (cf. Iversen et al. 2017; Guerrero‐Ramírez et al. 2021); consequently, root traits are less represented in functional trait literature.

Rooting depth is one dimension of the plant's occupancy in the soil space determining the potential pool of available resources (Casper and Jackson 1997; Palacio et al. 2017; Wang et al. 2018; Zhou et al. 2020). Although depth and distribution of root systems is in the focus of biome‐scale studies (e.g., Canadell et al. 1996; Wang et al. 2018; Blume‐Werry et al. 2023), the use of rooting depth in community ecology studies is currently hampered by lack of data for many species. Generally, rooting depth well correlates with below‐ground biomass and with specific root length (SRL) and might be a good metric to express competitive ability (Berendse 1979; Pérez‐Harguindeguy et al. 2013). Furthermore, the different rooting depths of species within a community provide an opportunity to study niche segregation (Palacio et al. 2017; Cabal et al. 2024; Chen et al. 2024). Rooting depth can influence the species' ability for colonization (Ranđelović et al. 2024), drought tolerance (Padilla and Pugnaire 2007; Zhou et al. 2020) or transpiration rate (Li et al. 2022), and thus, possibly also affect the microclimate (Wright and Francia 2024). The vertical distribution of a root system affects the distribution of soil organic matter (Jobbágy and Jackson 2000; Poirier et al. 2018), which influences carbon cycling (Blume‐Werry et al. 2023) and has importance in the protection of soil against erosion (Reubens et al. 2007; Stokes et al. 2009).

Previous studies have mainly investigated rooting depth in crops (e.g., Manschadi et al. 1998; Li et al. 2022) or woody plants (e.g., Padilla and Pugnaire 2007; Zhou et al. 2020; Cabal et al. 2024). There are studies that use a proxy for rooting depth (Zhou et al. 2020), while others simulated factors influencing root development under controlled conditions (Li et al. 2022). Few studies have used species‐level rooting depth data collected in situ, although, as outlined in the previous paragraph, examining this trait could help us discuss some issues of the functioning of ecosystems.

In this article, we provide two sets of original maximum rooting depth records of herbaceous plants measured on herbarium specimens and individuals from the field. Our study was inspired by the question of whether rooting depth data obtained from herbarium specimens are realistic enough (i.e., similar to measurements from the field) to be used in ecological studies. Thus, we aimed at comparing the herbarium and field‐derived datasets with each other and with global data compiled from the TRY database. We were interested in (1) pairwise correlations between the average maximum rooting depth of included species in the three data sources, (2) how precisely maximum rooting depth measured in the field (most realistic) be predicted using herbarium material or database data (most heterogeneous), as well as (3) how precisely maximum rooting depth could be predicted for plants of different sizes?

## 2 Materials and Methods

### 2.1 Rooting Depth Data and Species Selection

The complete dataset used in this work comes from three sources: field and herbarium data collection (see details below), as well as the TRY database. On the one hand, the species pool that can be considered is limited by the current content of the TRY database. On the other hand, the range of herbarium specimens with roots is also limited. We selected 100 species from the common species pool of the herbarium and TRY dataset and sampled them in the field. Data on these 100 species were included in the model construction (“training dataset”). An additional 20 species from the herbarium and field dataset were used to test the models (“test dataset”). The taxa finally included were chosen to represent an approximately balanced proportion of annuals and perennials (48:52 in the training dataset, 10:10 in the test dataset), dicots and monocots (67:33 and 10:10, respectively), and plants of different sizes (6–110 cm and 6.5–130 cm) if possible.

**FIGURE 1:**
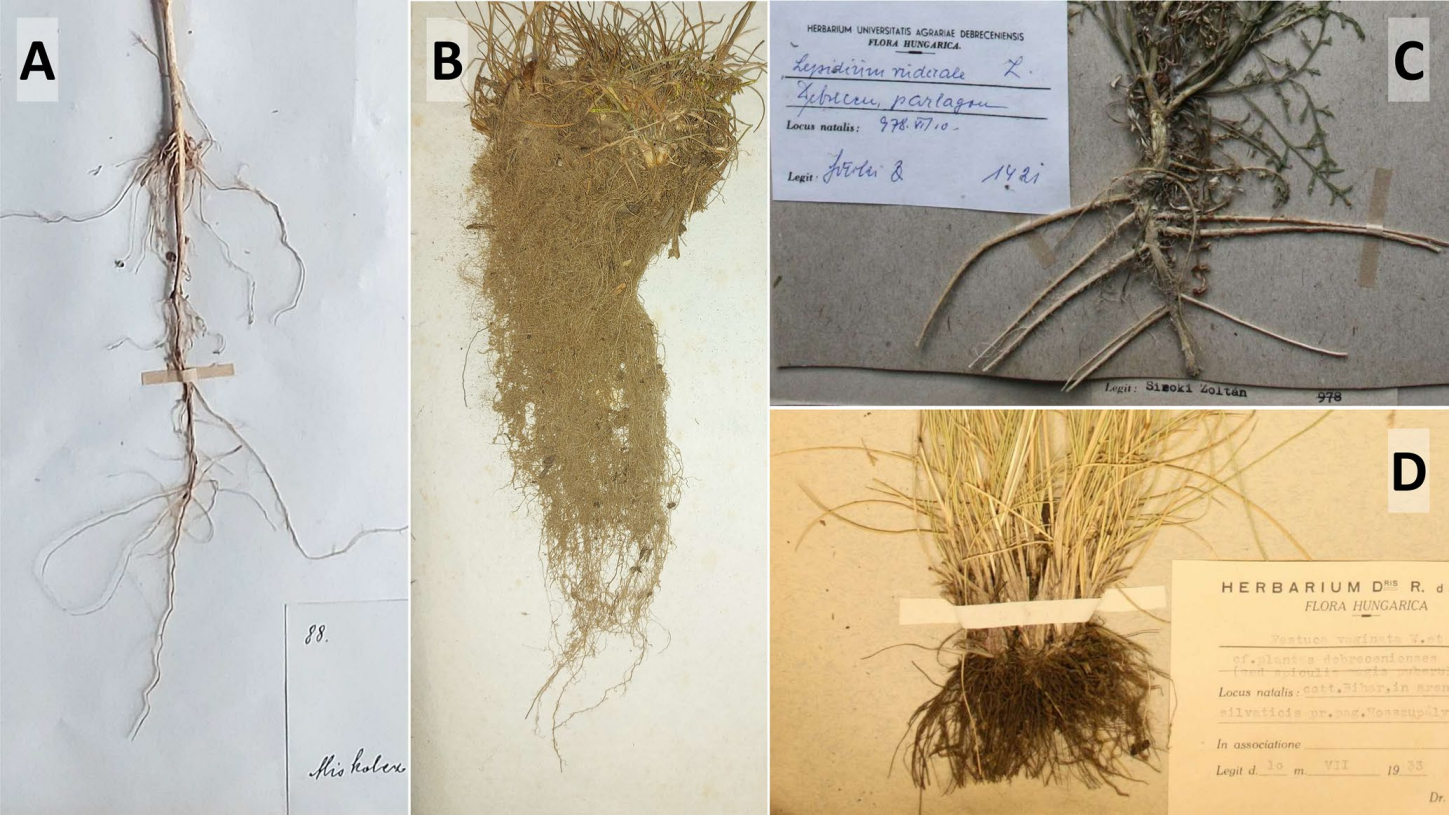
Examples for intact (A, B) and incomplete (C, D) taproots (A, C) and fibrous roots (B, D) of the surveyed herbarium specimens. Length of the intact roots was recorded as individual rooting depth data (A, C). Specimens with incomplete rooting systems were disregarded and excluded from further measurements (B, D).

**FIGURE 2:**
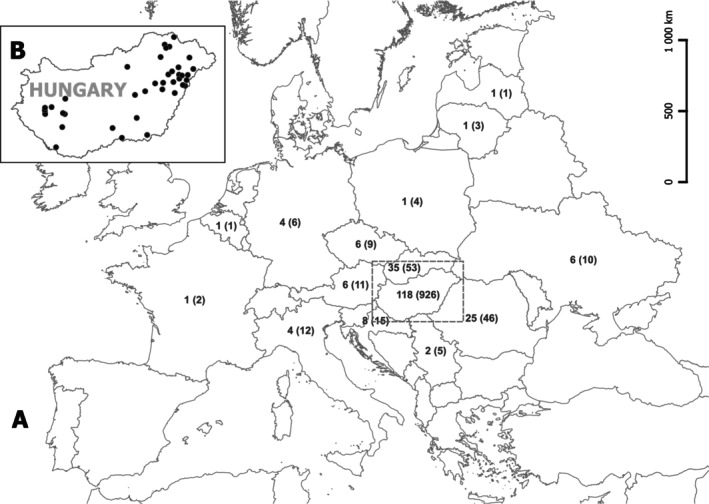
Spatial distribution of the processed samples. (A) Herbarium data. Number of taxa (and individual records in parentheses) by country. (B) Field data. Location of field sampling sites.

For 100 + 20 species, we surveyed the herbarium collection of the University of Debrecen (Debrecen, Hungary) in 2022 and the collection of the Hungarian Natural History Museum (Budapest, Hungary) in 2023. First, we reviewed the digital photocopy archive of the herbarium sheets searching for herbaceous vascular plant individuals which were collected and prepared with their roots. Second, we took out manually the sheets representing rooted individuals, and then, we selected those that appear to have complete root systems. We measured the vertical distance from the lowest point of the above‐ground shoot of the plants down to the deepest root tip (Figure 1). Individuals with damaged, incomplete, or abnormally arranged roots were excluded. Measurements were recorded individually by adding the species name and main collection data (place and date). The taxonomic identity of the processed specimens was thoroughly verified based on the most comprehensive identification key of the Hungarian flora (Király 2009). The used specimens originated from various European countries, with a Central European focus (Figure 2) and were collected between 1836 and 2023. The number of measurable individuals per sheet varied between 1 and 10. The number of measurements per species varied between 1 and 61 (Appendices S1 and S4).

Maximum rooting depth data for 100 + 20 species were collected from natural populations during 2022–2024 from various sites in Hungary (Figure 2; Appendix S2). We manually excavated root systems of adult plant individuals or ramets (in the case of clonally spreading species) in their entire vertical distribution. We made sure to reach the visible maximum rooting depth; however, some loss of the thinnest fine roots cannot be excluded (Freschet et al. 2021). Root systems reaching 0.5 m or deeper were examined on soil profile walls. Plant excavation sites were selected carefully, where the vertical spread of roots is unlimited by physical conditions (e.g., bedrock). We aimed to measure at least five individuals per species per population, but we increased the sample size where it was possible (Appendix S3). We considered these rooting depth measurements the most realistic ones.

To compare our original measurements to global standards, additional rooting depth data were obtained from the TRY database (Kattge et al. 2020) in 2022. Relevant data were accessible for 3668 species, 100 of which are represented in our original herbarium and field dataset. As rooting depth data are available for only a limited number of taxa, all values available in TRY were considered, regardless of their origin (estimated or measured) and methodological background (e.g., coring, excavation, trench wall).

### 2.2 Other Plant Traits

For each species with rooting depth data from the above‐mentioned sources, we compiled further plant traits and characteristics. We classified species according to their taxonomic position (monocots and dicots) (APG 2016), and life form (annuals [incl. therophytes and hemitherophytes] and perennials [incl. chamaephytes, hemicryptophytes, geophytes and hydato‐helophytes]) (Sonkoly et al. 2023). Plant height values were derived by averaging the minimum and maximum height values obtained from the Pannonian Database of Plant Traits (Sonkoly et al. 2023).

### 2.3 Data Analysis

All statistical analyses were performed in R v4.2.1 (R Core Team 2022). We averaged rooting depth data per species separately within the field, herbarium, and TRY datasets. To get approximately normally distributed rooting depth values, we applied natural base log‐transformation on field and herbarium data (field—Shapiro–Wilk's test: W = 0.99, *p* = 0.65, *n* = 100; herbarium—W = 0.99, *p* = 0.94, *n* = 100) (Shapiro and Wilk 1965; Pearson et al. 1977). However, although the best‐fitting distribution on values from TRY dataset was also estimated to be lognormal (“fitdistrplus” package, Delignette‐Muller and Dutang 2015; Akaike's Information Criterion (AIC)_lognormal_ = −15.90 vs. AIC_normal_ = 86.55), log‐transformation did not provide a normal distribution due to the large number of values exactly equal to 0.05 (*n* = 26) and extremely high values > 0.7 (*n* = 17). Therefore, we attempted to find the optimal normalization technique (“bestNormalize” package, Peterson and Cavanaugh 2020; Peterson 2021), which was given to be ordered quantile normalization. After applying this method on rooting depth values in the TRY dataset, we found that the best‐fitting distribution was indeed the normal (AIC_normal_ = 273.47, AIC_logistic_ = 278.50, AIC_uniform_ = 302.88, AIC_Cauchy_ = 319.55). Thus, we used the transformed values when rooting depth was entered as the response variable in further analyses.

First, we applied Pearson's correlation test to explore the relationship between pairwise comparisons of rooting depth data from the three sources. Second, we used rooting depth values from each of the three datasets as response variable in separated linear regression models and included life form, taxonomic position, and height as predictors. We added herbarium or TRY averages (separately) as new predictor in the model with field rooting depth response variable to evaluate their effect on model performance. We extracted Akaike's Information Criterion values corrected for small sample sizes (AICc, “AICcmodavg” package, Mazerolle 2020), *R* ^2^ values, and *F*‐statistics for each model. We also compared corresponding full and reduced models with likelihood ratio test available in the “lmtest” package (Zeileis and Hothorn 2002).

In the next step, we predicted maximum rooting depth values for all species in our datasets by applying a linear regression model, including measurements from the herbarium or TRY dataset, while also controlling for life form, taxonomic position, and species height. We repeated this step for predicting values for the “test dataset” to evaluate the performance of estimations, using herbarium data.

Finally, we assigned each species, based on measurements from the field, into one of the following categories considering soil horizons' thickness (Hartemink et al. 2020): shallow (< 0.2 m), medium (≥ 0.2 m and < 0.4 m), deep (≥ 0.4 m) rooting depth; and applied flexible discriminant analysis (FDA, “mda” package, Leisch et al. 2023) to check the correctness of this categorization using our mixed data (including categorical and continuous variables). We created all models with the possible combination of predictors, including herbarium or TRY source in separated sets. After this step, we calculated the root mean squared error (RMSE) using the predicted rooting depth values of species belonging to each category.

## 3 Results

Pearson's tests indicated that all rooting depth variables correlated significantly, but the strength of the relationships varied (Table 1). While measurements from the TRY database showed weaker correlation with data from the other two sources, values collected from herbarium were more strongly correlated to field measures.

**TABLE 1:** Pairwise correlation between rooting depth measures from different sources (*n* = 100).

	Herbarium	TRY
Field	0.66 ([0.53, 0.76]; *t* = 8.73)***	0.40 ([0.22, 0.56]; *t* = 4.36)***
Herbarium	—	0.26 ([0.06, 0.43], *t* = 2.64)*

*Note:* Values of 95% confidence interval and test statistics are shown in parentheses. Asterisks indicate the level of significance: * < 0.05, **< 0.01, *** < 0.001.

According to the linear regression models, life form, taxonomic position, and height could better explain the variance in rooting depth collected from either the field or the herbarium (Table 2); however, the model including TRY averages as the response variable performed weaker, showing only a significant effect of species' height. Considering that the rooting depth values measured in the field are the most accurate ones, we compared models with (full) or without (reduced) values from herbarium or TRY datasets as predictors, also controlling for the other properties (Table 3). The likelihood ratio tests confirmed that including herbarium data in the model can largely increase its performance, and thus, its predictive power, while the inclusion of TRY records contributes to the model to a lower extent.

**TABLE 2:** Summary of models including rooting depth (*n* = 100), from different sources, as response variable and its predictors.

#	Response variable	Predictors	Coefficient	SE	*t*	*p*	df	AICc	*R^2^*	F
1a	Field (log‐transformed)	Intercept	−2.39	0.09	−26.29	< 0.001***	96	128.25	0.31	16.06***
		Life form (annual vs. perennial)	0.47	0.09	5.00	< 0.001***				
		Taxonomical position (dicot vs. monocot)	−0.10	0.10	−0.98	0.331				
		Height	0.01	0.00	4.28	< 0.001***				
2	Herbarium (log‐transformed)	Intercept	−2.81	0.07	−38.15	< 0.001***	96	85.87	0.33	17.41***
		Life form (annual vs. perennial)	0.45	0.08	5.97	< 0.001***				
		Taxonomical position (dicot vs. monocot)	−0.31	0.08	−3.64	< 0.001***				
		Height	0.01	0.00	3.87	< 0.001***				
3	TRY (ordered quantile normalization)	Intercept	−0.58	0.18	−3.29	< 0.001***	96	260.21	0.15	7.05***
		Life form (annual vs. perennial)	−0.00	0.18	−0.02	0.985				
		Taxonomical position (dicot vs. monocot)	−0.14	0.20	−0.72	0.475				
		Height	0.02	0.00	4.52	< 0.001***				

*Note:* Asterisk indicates the level of significance: * < 0.05, ** < 0.01, *** < 0.001.

**TABLE 3:** Summary of models including field rooting depth (*n* = 100), as the response variable and its predictors, including measurements from either herbarium or TRY datasets.

#	Response variable	Predictors	Coefficient	SE	*t*	*p*	df	AICc	*R* ^2^	F	LRT
1b	Field (log‐transformed)	Intercept	−0.55	0.31	−1.77	0.080	95	97.52	0.50	25.86	*χ* ^2^ = 32.99***
		Herbarium (log‐transformed)	0.66	0.11	6.09	< 0.001***					
		Life form (annual vs. perennial)	0.17	0.09	1.84	0.069					
		Taxonomical position (dicot vs. monocot)	0.10	0.09	1.05	0.298					
		Height	0.01	0.00	2.44	0.017*					
1c	Field (log‐transformed)	Intercept	−2.01	0.13	−15.01	< 0.001***	95	117.26	0.39	16.97***	*χ* ^2^ = 13.25***
		TRY (log‐transformed)	0.17	0.05	3.67	< 0.001***					
		Life form (annual vs. perennial)	0.45	0.09	5.19	< 0.001***					
		Taxonomical position (dicot vs. monocot)	−0.08	0.10	−0.85	0.395					
		Height	0.01	0.00	2.52	0.014*					

*Note:* Asterisks indicate the level of significance: * < 0.05, ** < 0.01, *** < 0.001.

Model 1b (Table 3) provided the following equation for predicting average rooting depth values of species:

$$\begin{array}{c} \log\;\text{rooting depth}=-0.551+0.655^{*}\;\log\;\text{herbarium}\\ \;+0.005^{*}\;\text{height}\;\left(+0.171,\text{if perennial}\right)\;\\ \;\left(+0.099,\text{if monocot}\right) \end{array}$$

The equation is as follows based on Model 1c (Table 3):

$$\begin{array}{c} \log\;\text{rooting depth}=-2.014+0.165^{*}\;\log\;\mathrm{TRY}\\ \;+0.006^{*}\;\text{height}\;\left(+0.454,\text{if perennial}\right)\;\\ \;\left(-0.084,\text{if monocot}\right) \end{array}$$

**FIGURE 3:**
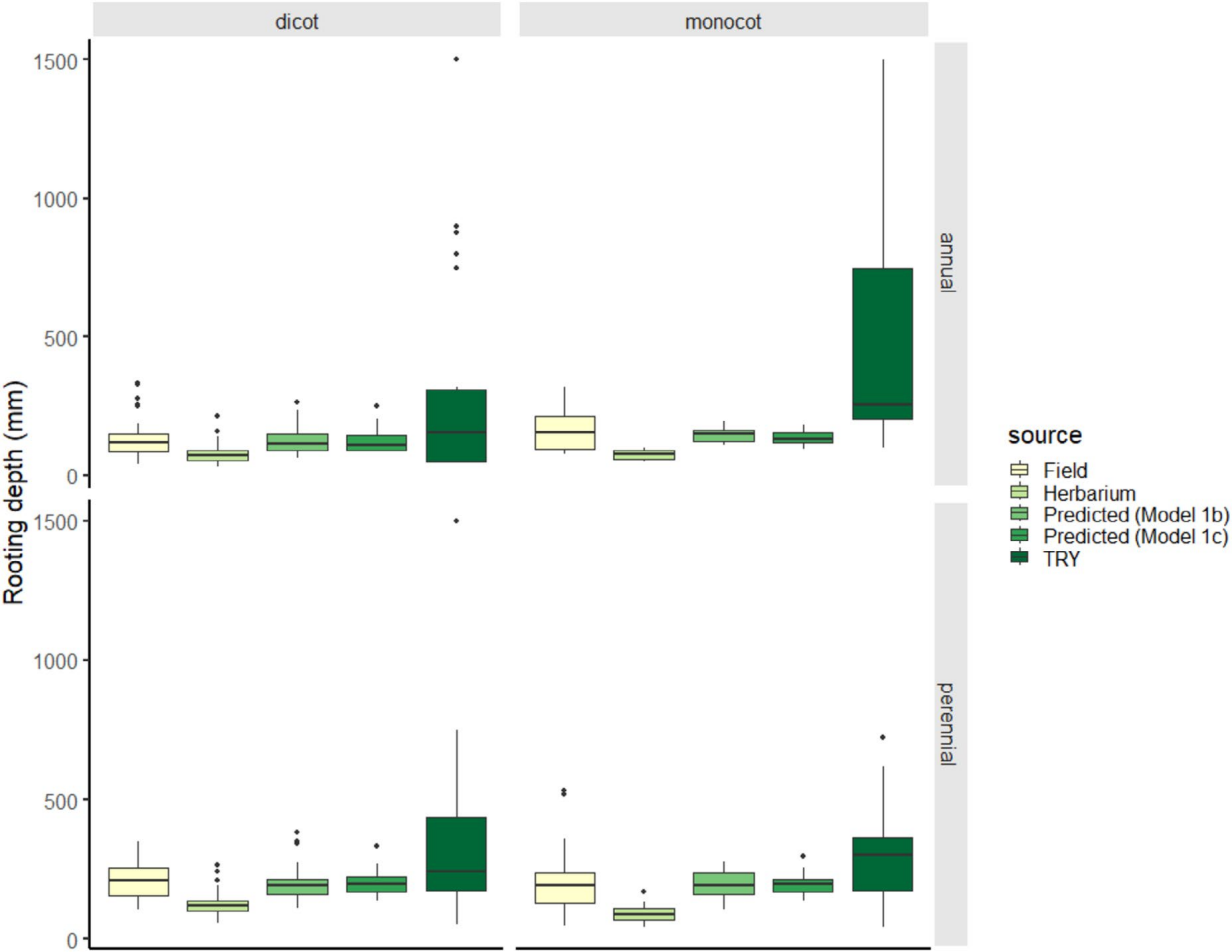
Comparison of rooting depth values (*n* = 100) obtained from different sources. Predicted values were estimated by the models, where Model 1b and Model 1c included herbarium and TRY measurements, respectively.

To obtain the average rooting depth values in meter, we need to calculate Euler's number raised to the power of the resulted values. Using the above models, we can predict the rooting depth of species (Figure 3) with an accuracy of 0.074 and 0.079 RMSE (root mean squared error, see also Appendix S4 for the predicted values), respectively. The predicted values (herbarium, mean ± SD: 0.166 ± 0.064 m; TRY, mean ± SD: 0.164 ± 0.055 m) are highly correlated with the original field measurements (0.177 ± 0.095 m) (herbarium: Pearson's *r* = 0.63, *t*_98_ = 8.11, *p* < 0.001, TRY: *r* = 0.57, *t*_98_ = 6.90, *p* < 0.001).

We applied Model 1b for estimating rooting depth values for an additional 20 species (“test dataset”). The model predicted rooting depth values for these additional 20 species well (0.059 RMSE), and the values correlated well with the corresponding field measurements (*r* = 0.74, *t*_19_ = 4.71, *p* < 0.001).

**FIGURE 4:**
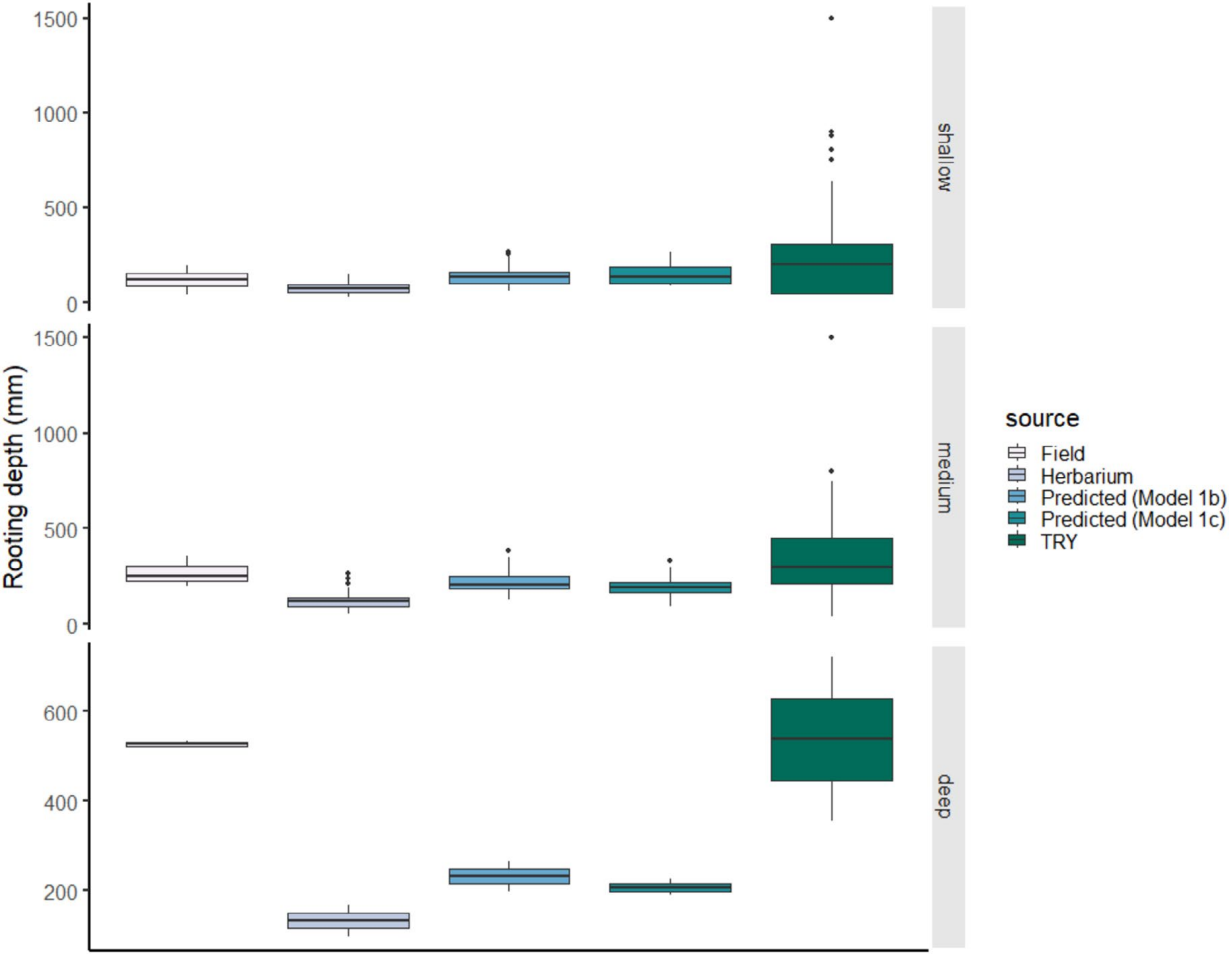
Comparison of rooting depth values (*n* = 100) obtained from different sources in three categories (shallow, medium, deep). Predicted values were estimated by the models, where Model 1b and Model 1c included herbarium and TRY measurements, respectively.

To further check the usefulness of herbarium and TRY sources, we applied flexible discriminant analysis on the depth categories (shallow, medium, deep rooting). We had 64, 34, and 2 species in the categories, respectively. The best model correctly identified the categories for 82% and 71% of the species when herbarium or TRY data were included, respectively (for more information on the FDA models see Appendix S5). The accuracy of the prediction for species in the three categories (shallow, medium, deep rooting depth, Figure 4) were 0.047, 0.083, and 0.297 RMSE with predicted values from herbarium measurements. It is worth noting here that only two species were in the deep rooting depth category, which number is insufficient for properly evaluating the accuracy of the predictions here, and thus, drawing any conclusion for this category would be misleading. We found similar accuracy values (0.050, 0.087, and 0.320 RMSE) for the predictions based on TRY measurements.

Finally, we emphasize that our presented dataset containing new rooting depth raw data for 120 vascular plant species (Appendices S1–, S4), for most of which only estimates have been available so far (cf. Kattge et al. 2020).

## 4 Discussion

The original intention of our study is to provide a new opportunity to extend the incomplete databases on root traits by including herbarium specimens. Roots have rarely been examined on herbarium material for several reasons. For instance, roots are often omitted from herbarium items (inconvenient size, difficulties during preparation, lack of taxonomic information, esthetic reasons, etc.). A large number of items have to be reviewed to find specimens with underground organs (cf. Younis et al. 2020). Moreover, trait data measured on herbarium material are usually considered biased and in need of correction (Queenborough and Porras 2014; Daru et al. 2018; Perez et al. 2020).

We tested the correlation between our field measurements, as realistic values, and herbarium‐derived datasets to provide information on the usability of herbarium data. The found strong correlation suggests that herbarium material can indeed provide reliable rooting depth records and can be considered a great resource of trait measurements, at least within certain limits.

We developed an equation to estimate a realistic rooting depth value of a given species based on the values measured on herbarium samples. The equation corrects the measured values based on the life form, taxonomic position, and average height of the plant and predicts an (average) maximum rooting depth value.

We found rooting depth across different taxa to be less variable in the two original datasets compared to the highly variable TRY dataset. Variance of the original datasets can be explained through additional properties. Life form, taxonomic position, and the above‐ground body size appear to be good predictors of rooting depth records, suggesting differences between annuals and perennials, dicots and monocots, and among plants of different sizes. Although the inclusion of some additional properties in the model limits its usability (due to the availability of suitable data), we believe that in the future it would be worthwhile to investigate the effect of additional, potentially predictive properties on rooting depth and improve the models by incorporating them. Root traits can of course not only be estimated from plant properties but also be influenced by environmental effects (e.g., soil structure, climate) that could be considered in future models. However, the complex effect of environmental factors on root development can be context‐dependent (Manschadi et al. 1998; Zhou et al. 2020; Cabal et al. 2024) and not yet sufficiently understood to incorporate these factors into predictions.

Records from the TRY database showed weaker correlation with the herbarium dataset than with the field measurements. In terms of variance in the TRY dataset, taxonomic position and life form had no effect, and only the plant height proved to be a good predictor of that variable. It is possible that the data taken from TRY are mostly estimates and that the original sources (e.g., Fitter and Peat 1994; Green 2009) simply assumed a deeper root system for larger plants. In general, it is not surprising to find differences between values from global datasets and local measurements due to differences in sampling methods, as well as differences in plant community dynamics, climate, and/or soil type (Schenk and Jackson 2002). Thus, the remarkable difference between our original datasets and the TRY dataset can be explained by several factors. First, there is a methodological heterogeneity of the records' primary sources underlying TRY, containing both measured and estimated rooting depth data. Estimates can be highly simplified and tend to provide information only on the relative sizes of the species included in a given source. The measured values were obtained following various procedures, such as excavation or coring to explore root systems. Secondly, plants grown in pots or cloth bags were also reported, although these values hardly represent the characteristics of plants growing in natural conditions. Finally, we must draw attention to the fact that some species are represented in the database by conspicuously incorrect rooting depth records. For example, for *Lamium amplexicaule* or *Arenaria serpyllifolia* (small‐sized annuals), the rooting depth > 1 m reported in the database is surprising—for both species, this estimated value is more than ten times greater than what we measured in the field, and this extreme difference can hardly be explained by environment‐induced infraspecific variability or methodological reasons. Because of this qualitative and methodological heterogeneity, the use of available trait data from such global databases requires careful evaluation, and a more standardized way of data measurement and reporting is suggested.

Based on our results, the use of dry‐stored plant material for root trait measurements is highly recommended. Data measured on herbarium specimens correlate more strongly with (presumably most accurate) values collected in the field than data obtained from global databases. However, the values measured on herbarium samples are generally lower than those measured in the field. This might be due to a combination of several factors. Besides the tissue shrinkage (Perez et al. 2020) and some fragmentation during drying and storage, we also have to consider potential biases related to the herbarium collection procedure. Herbarium collectors may not have attempted to excavate the entire root system, so it is possible (especially in case of fibrous root systems) that a specimen with apparently intact roots is missing the thinnest root sections that penetrated the deepest soil layers. During the collection of specimens, the selection of individuals from the population may have been biased (Schmidt‐Lebuhn et al. 2013), i.e., smaller individuals of tall herbs were preferred, as the plant needs to fit on a herbarium sheet of a certain size. This collection bias probably partly explains the difference between field and herbarium specimens of medium‐sized plants (Figure 4).

Furthermore, it should be mentioned that there may be some temporal bias in our dataset (we used averages of data from the 19th–21st centuries). Global changes are certainly affecting traits, but our knowledge in this area is still very incomplete (Moran et al. 2016). Many studies have documented shifts in species distributions and phenophases due to global warming. However, little is known about the morphological changes in organisms caused by climate change. In this aspect, Woodward's (1987) study was of pioneering importance: he showed that increased atmospheric carbon dioxide concentrations caused a decrease in leaf stomatal density. Several sources report on the temporal trends in changes of leaf traits (Guerin et al. 2012; Leger 2013; Li et al. 2020; Jaroszynska et al. 2023) and plant height (Leger 2013; Jaroszynska et al. 2023), which is explained by climate change. Global changes can also lead to rapid altering in flower traits, e.g., by selection for the ability to self‐fertilization (Cheptou et al. 2022; Austin et al. 2022). A perspective research direction would be to explore the possible time course of root traits of plant species (or guilds) through an extensive herbarium dataset.

The use of herbarium material as a source of trait records has several advantages that have been emphasized in a number of studies (Perez et al. 2020; Meineke and Daru 2021; Heberling 2022; Davis 2023). Here we highlight only a few aspects. Herbarium work is generally more time and cost‐effective than field work, mainly because the digitization of natural history collections (Soltis 2017) provides immediate access to a large number of taxa and specimens. Therefore, the carbon footprint of studies using already existing herbarium material is much lower than those generating new data. Herbarium material can be examined at a remarkable spatial (e.g., Aikio et al. 2010; Park et al. 2023) and temporal scale (e.g., Meineke et al. 2019; Jaroszynska et al. 2023); thus, the variability of a given character can be examined from both these perspectives. Root traits are often examined using destructive methods, which may cause the death of the studied individuals. Taking conservation into account, therefore, makes it difficult to collect data on rare, legally protected species. However, by studying museum collections, repeated destructive field sampling can be avoided, which is a more sustainable practice.

Nevertheless, there are limitations to the use of herbarium material in trait‐based studies (Daru et al. 2018) and in particular, root trait studies. Herbarium specimens contain mainly root systems of herbaceous plants as opposed to trees and shrubs, and complete root systems can only be studied for smaller or medium‐sized species with shallow or medium‐deep root systems (< 0.4 m).

Considering the advantages of trait‐based studies and the incompleteness of root trait data in global databases, we can recommend the widest possible use of herbarium data for root trait measurements. Further efforts to reveal and correct biases inherent in herbarium datasets should be similarly encouraged. Aggregation of herbarium specimen‐derived information into global databases can improve the completeness of these sources from spatial, temporal, and taxonomic points of view.

Finally, we must express our concern about the future of natural history collections. Although these collections, including herbaria, are typically appreciated as sources of scientific information, the professional maintenance of collections faces challenges (Dalton 2003). In the future, it is imperative that funders and users of scientific infrastructure allocate more resources to the maintenance, development, and curation of collections. Efforts should be made to involve the curators of the collections in future research and publication activities (Stern and Eriksson 1996). Otherwise, we can expect a dramatic decline in biodiversity research expertise (Páll‐Gergely et al. 2024). The irreplaceable wealth of information in the collections will no longer be able to grow, strict professional control may be lost, and the material may become inaccessible for researchers.

## Author Contributions

**Attila Takács:** conceptualization (equal), data curation (equal), methodology (equal), writing – original draft (equal). **Attila Molnár V.:** conceptualization (equal), data curation (equal), methodology (equal), writing – original draft (equal). **Anna E‐Vojtkó:** conceptualization (equal), methodology (equal), writing – original draft (equal). **Jenő Nagy:** conceptualization (equal), formal analysis (lead), methodology (equal), writing – original draft (equal).

## Conflicts of Interest

The authors declare no conflicts of interest.

## Supporting information


**Appendix S1.** Individually measured rooting depth records collected from herbarium specimens.

**Appendix S2.** Individually measured rooting depth records collected from field.

**Appendix S3.** Field records averaged by species.

**Appendix S4.** Herbarium records averaged by species, supplemented with predicted values.

**Appendix S5.** Summary of flexible discriminant analyses. K—number of predictors in the model, Correctness—the proportion of correctly identified categories by the model.

## Data Availability

We provide our data in appendices.
